# Supplementary material for: Impact of different frequencies of controlled breath and pressure-support levels during biphasic positive airway pressure ventilation on the lung and diaphragm in experimental mild acute respiratory distress syndrome
Source: PLoS One. 2021 Aug 20;16(8):e0256021. doi: 10.1371/journal.pone.0256021 (PMC8378704; doi:10.1371/journal.pone.0256021)
Supplement: S1 Table — (DOCX) [file pone.0256021.s001.docx]

**S1 Table: Forward and reverse oligonucleotide sequences of target gene primers**

| **Gene** | **Primer** | **Primer sequences (5′-3′)** |
| --- | --- | --- |
| ***Lung*** | | |
| **TNF-α** | **Forward** | **ACA AGC CCG TAG CCC ACG TC** |
|  | **Reverse** | **AGG AGC ACG TAG TCG GGG CA** |
| **CC-16** | **Forward** | **GAT CGC CAT CAC AAT CAG TG** |
|  | **Reverse** | **GGT ATC CAC CAG CCT CTT CA** |
| **VCAM-1** | **Forward** | **TGCACGGTCCCTAATGTGTA** |
|  | **Reverse** | **TGCCAATTTCCTCCCTTAAA** |
| **Amphiregulin** | **Forward** | **TTT CGC TGG CGC TCT CA** |
|  | **Reverse** | **TTC CAA CCC AGC TGC ATA ATG** |
| **Decorin** | **Forward** | **GAT CAG CCC AGA GGC ATT TA** |
|  | **Reverse** | **GCT CCA TTT TCA ATC CCA GA** |
| ***Diaphragm*** |  |  |
| **MURF-1** | **Forward** | **GGA TCA CTC AGG AGC AGG AG** |
|  | **Reverse** | **CTT GGC ACT CAA GAG GAA GG** |
| **MAFbx** | **Forward** | **TCA CAG CTC ACA TCC CTG AG** |
|  | **Reverse** | **GAC TTG CCG ACT CTC TGG AC** |
| ***36B4*** | **Forward** | **GGA TCA CTC AGG AGC AGG AG** |
|  | **Reverse** | **CTT GGC ACT CAA GAG GAA GG** |

***36B4*, acidic ribosomal phosphoprotein P0.**
