# Supplementary material for: Impact of different frequencies of controlled breath and pressure-support levels during biphasic positive airway pressure ventilation on the lung and diaphragm in experimental mild acute respiratory distress syndrome
Source: PLoS One. 2021 Aug 20;16(8):e0256021. doi: 10.1371/journal.pone.0256021 (PMC8378704; doi:10.1371/journal.pone.0256021)
Supplement: S2 Table — (DOCX) [file pone.0256021.s002.docx]

**S2 Table - Mean Arterial Pressure, and arterial blood gases at BASELINE-ZEEP**

| **Parameters** | **BIVENT-100+ PSV_0%_** | **BIVENT-50** | | | |
| --- | --- | --- | --- | --- | --- |
|  |  | **BIVENT-50+PSV_0%_** | **BIVENT-50+PSV_50%_** | **BIVENT-50+PSV_100%_** | |
| **MAP (mmHg)** | 140 ± 22 | 143 ± 27 | 123 ± 13 | 141 ± 19 | |
| **Arterial blood gases** | | | | | |
| **PaO_2_/FiO_2_ (mmHg)** | 260 ± 90 | 255 ± 69 | 271 ± 64 | | 252 ± 95 |
| **pHa** | 7.49 ± 0.06 | 7.49 ± 0.05 | 7.47 ± 0.08 | | 7.47 ± 0.04 |
| **PaCO_2_ (mmHg)** | 31 ± 3 | 27 ± 6 | 28 ± 4 | | 32 ± 2 |
| **HCO_3_^-^ (mmol/l)** | 24 ± 3 | 20 ± 4 | 20 ± 5 | | 23 ± 3 |

Values are given as mean ± standard deviation (SD) of 8 animals in each group. Comparisons between BIVENT-100 and BIVENT-50 groups were done using Student t-test (p<0.05). Comparisons among BIVENT-50 groups were done using One-Way ANOVA followed by Holm-Šídák post hoc test (p<0.05). MAP: mean arterial pressure; PaO_2_/ FiO_2_ = the ratio of arterial partial pressure of oxygen and fraction of inspired oxygen; pHa = arterial pH, PaCO_2_ = arterial partial pressure of carbon dioxide, HCO_3_^-^ = Bicarbonate. BIVENT: biphasic positive airway pressure at different rates of time-cycled controlled breaths (100 and 50 breaths/min); PSV_0%_: no pressure support ventilation; PSV_50%_: pressure support ventilation 50% P_high_; PSV_100%_: pressure support ventilation 100% P_high_; P_high_=spontaneous breaths at high continuous positive airway pressure.
