# Supplementary material for: Impact of different frequencies of controlled breath and pressure-support levels during biphasic positive airway pressure ventilation on the lung and diaphragm in experimental mild acute respiratory distress syndrome
Source: PLoS One. 2021 Aug 20;16(8):e0256021. doi: 10.1371/journal.pone.0256021 (PMC8378704; doi:10.1371/journal.pone.0256021)
Supplement: S3 Table — (DOCX) [file pone.0256021.s003.docx]

**S3 Table - Mean Arterial Pressure, the amount of fluids, and arterial blood gases at FINAL**

| **Parameters** | **BIVENT-100+PSV_0%_** | **BIVENT-50** | | | |
| --- | --- | --- | --- | --- | --- |
|  |  | **BIVENT-50+PSV_0%_** | **BIVENT-50+PSV_50%_** | **BIVENT-50+PSV_100%_** | |
| **MAP (mmHg)** | 123 ± 17 | 119 ± 30 | 116 ± 27 | 127 ± 15 | |
| **Fluids (ml)** | 5.2 ± 1.3 | 5.2 ± 2.5 | 6.1 ± 2.4 | 6.8 ± 2.3 | |
| **Arterial blood gases** | | | | | |
| **PaO_2_/FiO_2_ (mmHg)** | 305 ± 84 | 337 ± 57 | 391 ± 30 | | 329 ± 58 |
| **pHa** | 7.37 ± 0.12 | 7.36 ± 0.10 | 7.35 ± 0.08 | | 7.34 ± 0.05 |
| **PaCO_2_ (mmHg)** | 37 ± 4 | 40 ± 5 | 43 ± 7 | | 43 ± 8 |
| **HCO_3_^-^ (mmol/l)** | 24 ± 2 | 22 ± 3 | 21 ± 2 | | 23 ± 3 |

Values are given as mean ± standard deviation (SD) of 8 animals in each group. Comparisons between BIVENT-100 + PSV_0%_ and BIVENT-50 + PSV_0%_ groups were done using Student t-test (p<0.05). **vs*. BIVENT-100. Comparisons among BIVENT-50 groups were done using One-Way ANOVA followed by Holm-Šídák post hoc test (p<0.05), # vs BIVENT-50 + PSV_0%_; † vs BIVENT-50 + PSV_50%_.

MAP: mean arterial pressure; PaO_2_/ FiO_2_ = the ratio of arterial partial pressure of oxygen and fraction of inspired oxygen; pHa = arterial pH, PaCO_2_ = arterial partial pressure of carbon dioxide, HCO_3_^-^ = Bicarbonate
