# Supplementary material for: Impact of different frequencies of controlled breath and pressure-support levels during biphasic positive airway pressure ventilation on the lung and diaphragm in experimental mild acute respiratory distress syndrome
Source: PLoS One. 2021 Aug 20;16(8):e0256021. doi: 10.1371/journal.pone.0256021 (PMC8378704; doi:10.1371/journal.pone.0256021)
Supplement: S4 Table — (DOCX) [file pone.0256021.s004.docx]

**S4 Table - Respiratory Parameters adjusted at Baseline-ZEEP, INITIAL and FINAL**

|  |  | BIVENT-100 + PSV_0%_ | BIVENT-50 | | |
| --- | --- | --- | --- | --- | --- |
| Parameter | **Timepoint** |  | **BIVENT-50 + PSV_0%_** | **BIVENT- 50 + PSV_50%_** | **BIVENT-50 + PSV_100%_** |
|  | Baseline ZEEP | 8.0 ± 1.4 | 7.7 ± 0.7 | 8.3 ± 1.3 | 7.4 ± 1.2 |
| Adjusted Phigh (cmH_2_O) | INITIAL | 8.5 ± 2.5 | 7.8 ± 0.8 | 8.5 ± 1.4 | 8.1 ± 1.3 |
|  | FINAL | 12.8 ± 2.5 | 12.6 ± 1.3 | 13.2 ± 1.7 | 12.5 ± 1.9 |
|  | Baseline ZEEP | - | - | - | - |
| Adjusted PSV  (cmH_2_O) | INITIAL | - | - | 8.5 ± 1.4 | 8.1 ± 1.3 |
|  | FINAL | - | - | 4.6 ± 1.9 | 7.3 ± 2.0† |

Values are given as mean ± standard deviation (SD) of 8 animals in each group. Comparisons between BIVENT-100**+**PSV_0%_ and BIVENT-50**+**PSV_0%_ groups were done using Student t-test (p<0.05). Comparisons among BIVENT-50 groups were done using One-Way ANOVA followed by Holm-Šídák post hoc test (p<0.05); † *vs* BIVENT-50+PSV50%. BIVENT: biphasic positive airway pressure at different rates of time-cycled controlled breaths (100 and 50 breaths/min); PSV_0%_: no pressure support ventilation; PSV_50%_: pressure support ventilation 50% P_high_; PSV_100%_: pressure support ventilation 100% P_high_; P_high_=spontaneous breaths at high continuous positive airway pressure.
