# Supplementary material for: Impact of different frequencies of controlled breath and pressure-support levels during biphasic positive airway pressure ventilation on the lung and diaphragm in experimental mild acute respiratory distress syndrome
Source: PLoS One. 2021 Aug 20;16(8):e0256021. doi: 10.1371/journal.pone.0256021 (PMC8378704; doi:10.1371/journal.pone.0256021)
Supplement: S1 File — (DOCX) [file pone.0256021.s005.docx]

**S5.** **Custom-made software written in LabVIEW and routine written in MATLAB for data analysis (link to a permanent URL)**

<https://drive.google.com/drive/folders/1c6DWghQEcA5A0_ptb9OfOduKsWe8Mh2C?usp=sharing>
